# Disparity Between Punitive Attitudes Toward Stranger Rape and Partner Rape: Evidence From Cross-National Survey Data

**DOI:** 10.1177/08862605241307618

**Published:** 2024-12-31

**Authors:** Andrzej Uhl, Andrzej Porębski, Ewa Ilczuk

**Affiliations:** 1 University of Cambridge, Cambridge, UK; 2 Jagiellonian University, Kraków, Poland

**Keywords:** punitiveness, rape, partner rape, rape myths, rape perception, public opinion

## Abstract

While legally the same, acts of sexual abuse within and outside intimate relationships are not seen as equal by the public, and this distinction might also be reflected in preferred criminal punishment; some people might deem partner rape as deserving less harsh punishment than the rape of a stranger. Our secondary analysis examines differential punitiveness toward these two types of rape among the respondents (*n* = 11,383) to a large population survey conducted in 2021 in Austria, Czechia, Germany, Hungary, Poland, and Slovakia. As part of the survey, respondents chose preferred sentences for partner and stranger rape. Using these sentencing questions, we investigate the direction, extent, and demographic distribution of the differential punitiveness toward stranger and partner rape. A large group of respondents (ranging from 31.5% in Austria to 47.3% in Czechia) granted greater leniency to partner rape than to stranger rape and the reverse was rarely observed. More severe sentences were chosen for stranger rape more often than for partner rape. The individual bias toward leniency for partner rape was also typically stronger than the rare bias for stranger rape (the difference of 36 vs. 24 months of imprisonment, respectively). Relative leniency toward partner rape was particularly pronounced in Slavic countries, more prevalent among men, and positively correlated with age, right-wing authoritarianism, social dominance orientation and rape myth acceptance. Drawing on our results and previous scholarship, we attribute the observed disparities to the persistence of rape myths and the legitimation of intra-relationship sexual violence by conventional belief systems.

## Introduction

The public are highly punitive toward sexual offenders in general, but various extralegal factors influence how harshly people judge certain types of rape (Harper et al., 2017; Pickett et al., 2013; Pratt, 2000). Stereotypical beliefs about sexual violence may account for disparities in its assessment by the public regardless of the actual harm or blameworthiness (Socia et al., 2021). One of the major disparities is found between stranger rape and partner or marital rape, since “the relationship between perpetrator and victim is a critical component in public attitudes toward rape” (Basile, 2002, p. 341). Despite its devastating consequences for the victims (see Bennice & Resick, 2003 for review), intimate partner rape was downplayed or seen as a private matter between partners and “an extreme extension of traditional male-female sexual interaction” (Bridges, 1991, p. 292). The laws of Germany, for instance, exempted forcible marital intercourse from the statutory definition of rape until 1997. Rarely do public morals change overnight as a result of new legislation, and citizens might still judge partner and stranger rape differently.

This study examines whether the erstwhile distinction between partner and stranger rape is also reflected in disparate punishment preferences. Are the public willing to afford greater leniency for partner rape? We also assess the prevalence (how many people show bias^1^ in favor of partner rape) and the size of that disparity (how much lighter they want the punishment for partner rape to be). Our hypothesis is that the disparity in sentences recommended for stranger and partner rape will reflect the spread of popular stereotypes about sexual violence. We, therefore, ask if, and how well, the acceptance of rape myths and their sociopolitical covariates can predict disparate punishment preferences for stranger and partner rape (“differential punitiveness”).

To answer these questions, we perform a secondary analysis of representative survey data gathered in Germany, Poland, Czechia, Austria, Slovakia, and Hungary for the 2021 Central European Social Survey (CESS). We thus attempt to extend the research on punitive attitudes beyond the English-speaking countries. A precise operationalization of punishment preferences—sentencing questions informed by penal law in force—is another strength of the survey. Nationally representative samples, which are seldom obtained in this line of research, allow us to trace the observed differences back to major social forces within the studied populations, spanning religion, politics, and culture.

## Literature Review

### Rape Myths and Partner Rape

A body of research confirms the tendency to treat rape perpetrators with whom the victim had a close relationship more leniently than those who were strangers, and these findings hold true in studies on marital, acquaintance, date, and partner rape (e.g., Ben-David & Schneider, 2005; Cowan, 2000; Osborn et al., 2021; Schwarz et al., 2022; Viki et al., 2004). To understand the basis of this differential punitiveness, it is essential to examine both the judgments and beliefs about sexual abuse, as well as how they are embedded within cultural frameworks and personal worldviews.

To begin with, we may ask what people usually mean when they say “rape.” Researchers have identified the so-called “real rape scenario”—a folk concept including beliefs and intuitions about how rape typically happens (Du Mont et al., 2003). According to this scenario, rape is an act of violent, forcible sexual penetration by a strange male assaulter in a public, deserted place, while the victim is portrayed as a resisting, morally upright white woman who consequently sustains physical and psychological injury (e.g., Krahé et al., 2007; Littleton & Axsom, 2003; Williams, 1984). Like many other forms of rape (e.g., prostitution rape, lesbian rape), partner rape does not match this description. Survey studies show that for many respondents, rape in marriage is viewed as highly unlikely, if not impossible (Boakye, 2009; Kirkwood & Cecil, 2001). If considered rape at all, it is believed to occur accidentally, with little premeditation (Kuriakose, 2019), or even to result from a misunderstanding (e.g., Bridges & McGrail, 1989). Additionally, victims of marital rape are more often blamed for the rape (Monson et al., 1996, 2000). As regards the current research question, each of these stereotypes is likely to blunt the punitive reaction toward a partner relative to stranger rape.

These stereotypes belong to a broader set of beliefs known in the literature as *rape myths*. Burt (1980, p. 217) defined them as “prejudicial, stereotyped, or false beliefs about rape, rape victims, and rapists” and presented a non-exhaustive list, to which the partner rape myths were later added (Payne et al., 1999). A more recent definition comes from Lonsway and Fitzgerald (1994, p. 134), according to whom rape myths are “attitudes and beliefs that are generally false but are widely and persistently held, and that serve to deny and justify male sexual aggression against women.” The key issue is not the myths’ accuracy in a particular situation, but rather how they “tend to be universally applied, as echoed in jury verdicts, public policy decisions, and personal reactions to survivors of sexual violence” (Lonsway & Fitzgerald, 1994, p. 135). Rape myths include statements such as “women want to be raped” or “women lie about rape,” but of particular relevance to the current research are those concerning the victim-perpetrator relationship.

At the heart of the *partner rape myth* lies the denial of the revocable nature of sexual consent. According to the extreme variant of the myth, a woman entering a romantic relationship expresses a lasting agreement to sexual activity between partners. For marital rape, this is strengthened by the idea of “conjugal duties” allegedly owed by the wife to the husband from the nuptials onwards (Kaganas & Murray, 1983). There are also “milder” forms of this myth. For instance, victims’ suffering is considered lesser if they consented in the past and it is assumed that the act occurred in a less violent way (Simonson & Subich, 1999).

### Correlates of Rape Myths Acceptance

Are some people more likely than others to subscribe to such beliefs? In the search for the predictors of differential punitiveness toward stranger and partner rape, we choose to rely on rape myth acceptance in general and its known correlates. Previous studies have consistently found men to be more supportive of rape myths than women (Barnett et al., 2018; Burt, 1980; Johnson et al., 1997), including the myths dealing specifically with marital rape (Ewoldt et al., 2000; Kirkwood & Cecil, 2001). Regarding age and occupation, research yields inconclusive results (Lonsway & Fitzgerald, 1994); these variables as such might have no direct relationship with rape myth acceptance but may correlate with other relevant variables (e.g., education and religiosity).

Thus, Fansher and Zedaker (2022) contend that the decisive predictors of rape myth acceptance are found not among demographic variables, but rather in strong adherence to traditional belief systems. Indeed, research shows a link between belief in rape myths and various attitudes associated with a conservative worldview, especially conservative gender role ideology (Johnson et al., 1997) and religiosity. In the Southern US, students identifying as Protestants or Roman Catholics exhibited higher levels of rape myth acceptance than agnostics and atheists (Barnett et al., 2018), and the levels of religiosity and religious intolerance also showed a positive relationship with rape myth acceptance (Aosved & Long, 2006).

Further known predictors of rape myth acceptance are right-wing authoritarianism (RWA) and social dominance orientation (SDO; Nicol & Tóth-Király, 2024; Süssenbach & Bohner, 2011). RWA items express beliefs in coercive social control, respect for existing authorities, and conforming to traditional moral and religious norms and values (Duckitt et al., 2010). Social dominance can be described as a “general attitudinal orientation toward intergroup relations, reflecting whether one generally prefers such relations to be equal, versus hierarchical” and the “extent to which one desires that one’s ingroup dominate and be superior to outgroups” (Pratto et al., 1994, p. 742).

Based on this cursory review of factors linked to rape myth acceptance, we have identified potential characteristics of people who might be more likely to judge partner rape less harshly than stranger rape. These characteristics are, among others, age, gender, religion, and authoritarian personality. We will later use them as hypothesized predictors of differential punitiveness toward stranger and partner rape. Before reporting our results, let us now turn to the prior studies that document such differential punitiveness.

### Differential Punitiveness Toward Stranger and Partner Rape

How is the distinction between stranger and partner rape borne out in research with a focus on punishment? A handful of studies investigated partner rape in the context of punitive sentiments but, in most cases, punitive responses feature as a secondary measurement of broader concepts such as rape myth acceptance. Consistently, punishment recommendations for rape have been found to increase as the relationship between the perpetrator and the victim becomes more distant, ranging from a total stranger, through neighbor, acquaintance, and date, to a romantic partner or spouse (Ben-David & Schneider, 2005; Cowan, 2000; Viki et al., 2004).^2^ The recent experiment by Osborn et al. (2021) confirmed this tendency in the consequential context of juror decision-making. Other studies have found that partner rape was more often attributed to female precipitation rather than to misogyny or male pathology (Cowan, 2000), and portrayed as less damaging (Ben-David & Schneider, 2005), suggesting that these views mitigate recommended punishment.

Although men and women as well as people of different ethnicities vary in the declared attribution of rape (Cowan, 2000), most studies do not report whether the disparity between punishment recommendations for partner and stranger rape differed across variables other than the hypothetical scenario itself. One relevant moderation effect was observed by Viki et al. (2004) in a small sample of British students; while the type of rape had a strong main effect on punishment, benevolent sexism only influenced the sentence length in an interaction term with the rape type; benevolent sexists were harsher, on average, toward rape in general but were exceptionally lenient toward acquaintance rape. Only recently has a conjoint experiment by Schwarz et al. (2022) shown the differential treatment of stranger rape versus acquaintance rape to also be contingent on political ideology. Conservatives were more likely to recommend a case featuring a stranger for harsher punishment, while no such effect was observed among moderates and liberals.

The current state of research on the public’s differential punitiveness toward rape is geographically diverse and compares a variety of scenarios (Ben-David & Schneider, 2005; Cowan, 2000; Viki et al., 2004). Nonetheless, the median sample size in the reviewed studies is low at 294, with most samples drawn from student populations, often from a single institution. Such samples might preclude some insights given the anticipated link between differential punitiveness and rape myth acceptance—a phenomenon known to be non-randomly distributed among age groups and social strata (see above). We agree with Ben-David and Schneider (2005, p. 397) that “future researchers should include participants from a wider range of age and education levels.” Additionally, the current study is the first work in this line of research to offer a cross-national comparison.

Moreover, the previously employed measures of punitiveness often fail to provide accurate estimates of preferred punishments and frequently diverge from the social realities of the justice system. For instance, the potential jurors surveyed by Osborn et al. (2021) could only impose a sentence of up to 10 years while the true maximum penalty for rape in England and Wales reached even 19 years (Sentencing Council, 2023). Elsewhere, researchers employed huge intervals (“1–3 years in jail”; Cowan, 2000), or used vague statements (“very severe punishment”; Ben-David & Schneider, 2005). What a respondent sees as “very severe” depends not only on how they understood this phrase *in abstracto* but also on their view of the offense itself. For instance, a fairly short prison sentence may strike one as very harsh for a crime that person sees as rather petty. Therefore, unmeasured factors could have influenced responses in unpredictable ways.

Lastly, we propose to investigate the moderation effects on the relationship between the type of rape (partner/stranger) and punitiveness. Previous studies established a difference between types of rape, but we do not know yet how this relationship differs across gender, nationality, and other salient characteristics. This is because student samples are not sufficiently diverse to determine whether the differential punitiveness occurs only in some social groups or in society as a whole.

Our study attempts to advance this line of research by investigating not only whether the bias in favor of partner rape exists, but also who is more likely to exhibit it, and how large it is. For more valid and generalizable insights, we conduct a secondary analysis on a cross-national, representative sample, and use specific measures of punishment preferences informed by legal provisions.

## Methods

### Sample and Procedure

The sample originates from the first wave of CESS (*n* = 11,383), with data collection spanning December 2021 and January 2022. Following approval by the research ethics committee for social sciences at the University of Warsaw (Decision no. 15/21), commercial contractors carried out the online survey in samples drawn from large panels of respondents. For each country, cross-quotas were set for gender and age categories, and simple quotas for the community size. The sample was then randomly selected from the panels with the size adjusted by the assumed level of the response rate. Sampled respondents were recruited through email invitations. The response rate varied from 10% in Germany to 59% in Czechia. The number of completed questionnaires was 1,630 in Czechia (mean age = 50.0, 48.8% male), 1,646 in Slovakia (mean age = 47.3, 49.5% male), 1,721 in Hungary (mean age = 49.3, 48.3% male), 1,901 in Poland (mean age = 47.1, 48.4% male), 2,220 in Germany (mean age = 52.4, 48.2% male), and 2,265 in Austria (mean age = 50.1, 49.7% male). The average interview length was 33 minutes. The survey consisted of several modules, concerned with a variety of social, political, and economic issues (CESS, 2023).

As part of the criminological module (Ostaszewski et al., 2024), the respondents were asked to choose a penalty they would recommend for first-time offenders found guilty of a given crime. Each study subject responded to the same set of five offenses in the following order: street assault, domestic assault, stranger rape, partner rape, child support payment evasion. The exact phrasing was: “In your opinion, what punishment should be imposed on a first-time offender who has committed the following act: (. . .) 3. Raping a stranger; 4. Raping a partner (. . .).”

The list and order of available penalties were modeled on a criminal code that was highly typical of the region. Alongside the statutory penalties such as a fine, community service, suspended prison sentence with or without supervision by a probation officer, and an unconditional prison term, a respondent could manually enter another penalty. The options were presented to the respondents in the same order. Although some can deem a fine more severe than a suspended prison sentence, we lack relevant empirical evidence in this respect and have decided against diverging from the statutory ranking. Then, the respondents who selected the unsuspended custodial sentence determined its length, which ranged from “1 month” to “25 years or a life sentence,” an interval of 1 month.

### Measures

#### Dependent Variable: Relative Leniency Toward Partner Rape

To determine subjects’ relative punitiveness toward each offense, we computed a binary variable “relative leniency toward partner rape,” which assumed a positive value whenever a respondent chose a less harsh penalty for partner rape. For example, the variable assumed a positive value for a respondent who demanded a fine for partner rape, and a prison term for stranger rape, or if the duration of the chosen unsuspended sentence for stranger rape exceeded that of the unsuspended sentence for partner rape. A negative value was assigned to respondents who either chose equal penalties for stranger and partner rape or a harsher penalty for partner rape.

Regarding the manually entered “other” penalties, a detailed qualitative review of these textual inputs returned four aggregate categories: “death penalty,” “eye for an eye,” “castration,” and “corporal punishments,” the last three of which we subsumed under an umbrella term “inhumane penalties.” In creating the variable “Relative leniency toward partner rape” the death penalty was considered harsher than all other punishments, and castration and corporal punishment were considered harsher than suspended prison terms and lighter punishments. We did not compare imprisonment and inhumane penalties to avoid arbitrary judgment (we coded such cases as missing values of relative leniency).

#### Rape Myth Acceptance

We used responses to five rape myths items adapted from the larger Illinois Rape Myth Acceptance Scale (CESS, 2023; Payne et al., 1999). The adapted items were unrelated to partner or marital rape. The sample item was “When women go to parties dressed in provocative clothing, they are asking for trouble.”^3^ The responses ranged from 1–“strongly disagree” to 5–“strongly agree.” With one exception, the responses strongly loaded on the same factor (loadings ≥ 0.59, four items’ Cronbach’s α = .79), which justified the use of the factor based on the four remaining items (calculated using a regression method) as a standardized rape myth acceptance index.

#### Right-Wing Authoritarianism

RWA was measured by a nine-item scale adapted from Beierlein et al. (2014). The sample item is “Well-established norms of behavior should not be questioned.” The responses ranged from 1–“strongly disagree” to 5–“strongly agree.” The scale had good consistency (α = .85). The mean value was 3.21 (*SD* = 0.79).

#### Social Dominance Orientation

SDO was measured by an abridged five-item scale included in the survey after Bilewicz et al. (2017). The responses ranged from 1–“strongly disagree” to 5–“strongly agree.” The scale had acceptable consistency (α = .74). The mean value was 2.24 (*SD* = 0.78).

#### Demographic Variables

Since gender norms vary widely across countries, we included respective dummy variables together with further demographics. In line with previous findings (see literature review above), gender and age were expected to influence the dependent variable. As many of the studied societies are characterized by a sharp urban-rural cultural divide, the degree of urbanization was also controlled for. Educational achievement was measured in completed years of schooling (*M* = 14.21, *SD* = 3.18). Religion was coded as a series of binary variables, each for any denomination sufficiently represented in the sample (Catholics, Protestants, Orthodox Christians, and Muslims), with atheists being the reference category. Detailed information on the demographic characteristics of the sample is included in the survey documentation (CESS, 2023).

### Analytic Strategy

The analysis consists of four parts. The first part, titled “*direction of disparities*,” traces the entire dataset for disparities in the punishment of partner and stranger. It primarily employs visual analysis to examine whether either type of rape is typically subject to greater leniency. In the second part, we compare the *size of disparities* between two possible cases of preferential treatment. In the third part, concerning the *societal distribution of disparities*, three logistic regression models are estimated, determining which variables predict differential punitiveness. Lastly, as a part of the third model analysis, we examine *the effect of the rape myth acceptance* together with the changes it introduces to the model. In statistical modeling for the last two parts, we excluded the cases where the punishment for the stranger was the lower one since this group was narrow and qualitatively different from the group proposing equal punishment.

## Findings

Respondents overwhelmingly chose unsuspended prison sentences for both partner (72.7%) and stranger rape (82.9%). The mean proposed duration of this sentence was 108 months for partner rape (*SD* = 90.6, median = 71) and 115 months (*SD* = 90.4, median = 73) for stranger rape. The second most popular type of punishment was a suspended prison sentence with supervision; 13.5% of respondents chose it for partner rape and 5.7% chose it for stranger rape. With these absolute figures in mind, we will now turn to the severity of penalties chosen for these two crimes in relation to one another.

### Direction of Disparities

**Figure 1.**
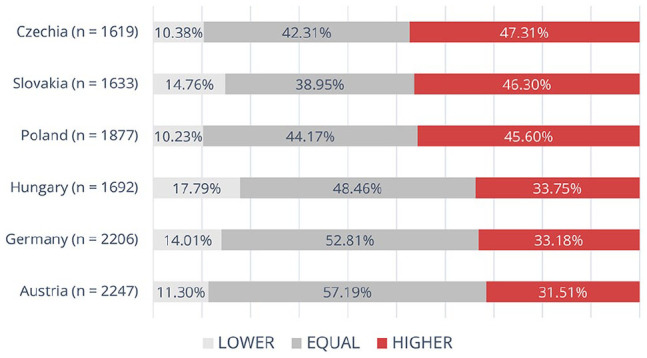
Recommended penalty for stranger rape in relation to partner rape penalty (by country). *Note.* “Higher” is the proportion of respondents who recommended a harsher penalty for stranger rape than for partner rape, “Lower” is the proportion of respondents who recommended the opposite, and “Equal” is the proportion of respondents who recommended the same punishment for stranger and partner rape.

The percentage of respondents proposing equal punishments for partner and stranger rape ranged between countries from about 39% for Slovakia to about 57% for Austria, see Figure 1. In Austria and Germany, more than half of the respondents proposed equal penalties, Hungary had a similar percentage of equal and different penalties, and in the remaining countries, different penalties were more frequent than equal. The proportion of subjects who recommended a higher punishment for stranger rape than for partner rape was larger than those who demanded more lenient treatment for stranger rape in every country, see Figure 1. There were significant differences between countries (*χ*²(10) = 290.370, *p* < .001), with the group treating partner rape more leniently being significantly larger in Czechia, Slovakia, and Poland relative to Hungary, Germany, and Austria.

**Figure 2.**
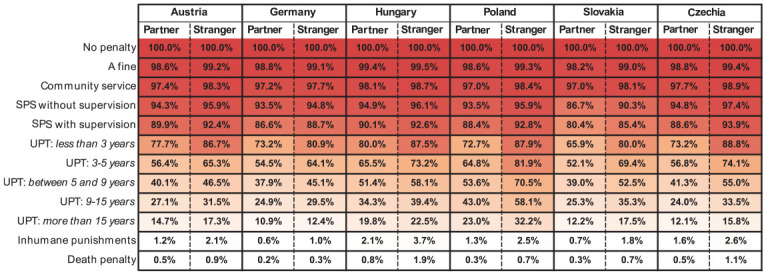
Heatmap of cumulative percent of penalty selections in increasing order for partner and stranger rape, among six countries. *Note.* Values in each row denote the fraction of people that selected a penalty from the row or harsher. The larger part of a column is colored, the higher is the punitiveness of the subject from a country toward a type of rape. SPS = suspended prison sentence; UPT = unconditional prison term.

Figure 2 extends the prior plot and presents the cumulative percent of specific penalty selection, split by countries and type of rape. Numbers indicate that *x*% of respondents chose a penalty displayed in the specific row or a harsher penalty, and red is more intense in fields with higher cumulative percent. The larger the red part of the column, the higher the punitiveness toward partner or stranger rape is in a given country. Two patterns emerge: firstly, in all countries, punitiveness toward stranger rape is higher in relation to partner rape; secondly, in Slovakia, Czechia, and Poland, differences between columns for stranger rape and partner rape are particularly pronounced.

**Figure 3.**
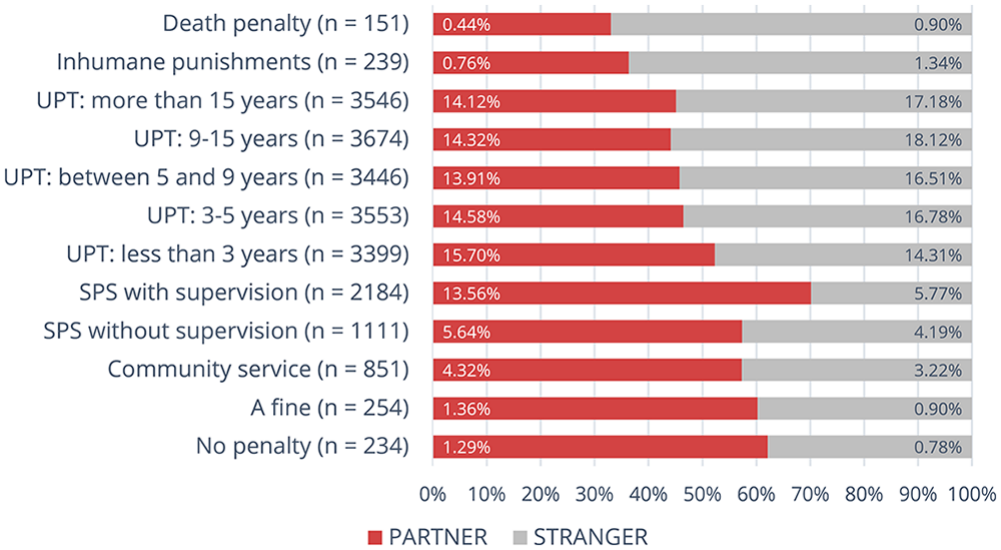
Distribution of particular penalties’ assignments to partner and stranger rape. *Note.* Values refer to the percent of penalty selections among all selections, separately for partner rape and stranger rape group (thus, values sum to 200%). *X* axis represents the proportion of selections as punishment for partner/stranger rape among all the selections for the punishment.

Figure 3 shows a clear, almost linear trend: more severe penalties were recommended more often for stranger rape and less often for partner rape. For example, of all recommended fines, 60% featured in the cases of partner rape, while 55% of cases of three or more years in prison involved stranger rape. The highest discrepancy can be observed in the death sentences (67% were recommended for stranger rape) and, interestingly, suspended prison sentences with supervision, recommended predominantly (70%) for partner rape, suggesting that respondents deemed this punishment particularly appropriate for partner rape.

### Size of the Disparities

To examine how much more leniently an average subject responded to partner rape, we compared median penalties recommended for either offense, see Table 1. Cross-country differences in disparities are evident (*p* of Kruskal–Wallis test < .001); in Poland, the difference between the median values was about 4 years, whereas in Austria the difference was just above 1 year. This tabulation, however, does not discriminate between those who favored partner rape from those more lenient toward stranger rape.

**Table 1.** Comparison of Median Penalty Recommendation (in Months of Imprisonment) by Type of Rape and Country.

Country	Partner Rape	Stranger Rape
Czechia	47	66
Slovakia	37	65
Poland	71	120
Hungary	61	71
Germany	41	60
Austria	47	60

*Note*. In each case, the median recommended penalty was an unsuspended prison sentence, but these sentences varied in duration. The exact values of that median penalty (cell values) are indicated in months of imprisonment.

**Figure 4.**
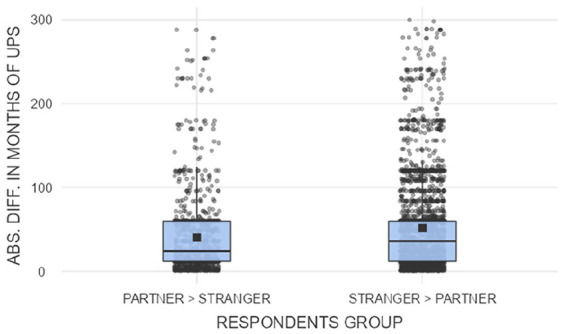
Box plots and jittered data presenting the distributions of difference between the proposed duration of an unconditional prison sentence (in months) in the group more lenient toward stranger rape (on the left, *n* = 1,019) and more lenient toward partner rape (on the right, *n* = 2,572). *Note.* UPS = unconditional prison sentence.

Figure 4 shows that the size of disparity measured in months of imprisonment was typically larger among those who granted greater leniency to partner rape. The comparison includes only the cases where prison term was recommended for both offenses, which allows for an interval measure of disparities and is warranted by the high number of people who selected custodial sentences for both stranger and partner rape (*n* = 7932, 69.68% of the sample). The median difference between sentences was 36 months among those favoring partner rape (IQR = 48, *M* = 51.59, *SD* = 55.25), as opposed to 24 months among those favoring stranger rape (IQR = 48, *M* = 40.23, *SD* = 49.71). The disparity in favor of partner rape was not only more common but also typically stronger.

### Societal Distribution of Disparities

Three logistic regression models were estimated. The first model (“COUNTRY”) included only the country variable and serves as a benchmark for the other two models, while the second model (“FULL”) included all predictors except Rape Myth Acceptance, specifically demographic variables, religion, and psychological scales. The third model (“FULL + RMA”) extended the second model by including Rape Myth Acceptance variable. The FULL model is presented in Table 2, while Table 3 includes measures of the models’ quality and their comparison. Moreover, Table 2 presents FULL + RMA *χ*² omnibus likelihood ratio test to allow conclusions about the effect that including Rape Myth Acceptance had on other variables. The models were estimated on a subsample excluding the heterogenous group that chose a lower punishment for stranger rape.

**Table 2.** Binomial Logistic Regression Models for “Relative Leniency Toward Partner” (1–Higher Proposed Penalty for Stranger; 0–Equal Penalties) as Response Variable.

Predictor	Odds ratio [95% CI]		*z*	*p*	Omnibus test *χ*²	
					FULL	FULL + RMA
Intercept	0.315	[0.258, 0.384]	−11.369	< .001	—	—
Country (ref.: Austria)					188.298	143.478
Czechia	2.047	[1.770, 2.367]	9.670	< .001		
Germany	1.080	[0.939, 1.241]	1.079	.280		
Hungary	1.249	[1.079, 1.445]	2.975	.003		
Poland	1.867	[1.624, 2.145]	8.793	< .001		
Slovakia	2.021	[1.745, 2.340]	9.405	< .001		
Male (ref.: female)	1.342	[1.234, 1.458]	6.909	< .001	47.894	31.882
Age (ref.: 18–24)					173.318	175.940
25–34	1.046	[0.874, 1.253]	0.490	.624		
35–44	0.965	[0.809, 1.151]	−0.397	.692		
45–54	1.079	[0.903, 1.288]	0.838	.402		
55–64	1.322	[1.107, 1.579]	3.089	.002		
65 and more	2.031	[1.708, 2.415]	8.024	< .001		
Years of education (standardized)	0.957	[0.917, 0.999]	−2.003	.045	4.021	1.356
Urbanization (ref.: rural areas)					2.390	1.953
Towns	1.059	[0.957, 1.172]	1.113	.266		
Cities	0.979	[0.884, 1.083]	−0.412	.680		
Religion (ref.: none/atheist)					37.970	29.019
Catholic	1.308	[1.183, 1.445]	5.248	< .001		
Muslim	1.732	[1.212, 2.477]	3.014	.003		
Protestant	1.359	[1.158, 1.595]	3.762	< .001		
Orthodox	1.350	[0.911, 2.001]	1.495	.135		
Other	1.388	[1.068, 1.804]	2.451	.014		
SDO (standardized)	1.115	[1.065, 1.166]	4.686	< .001	21.998	4.221
RWA (standardized)	1.055	[1.008, 1.104]	2.297	.022	5.279	0.511

*Note*. RMA = rape myth acceptance; RWA = right-wing authoritarianism; SDO = social dominance orientation.

**Table 3.** Binomial Logistic Regression Models Quality Measures and Comparison.

Model	AIC	*R*²_McF_	*R*²_CS_	*R*²_N_	*χ*²	*df*	*p*
(1) COUNTRY	13265.53	.016	.022	.030	220.144	5	< .001
(2) FULL (without RMA)	12978.49	.040	.054	.072	539.181	21	< .001
(3) FULL + RMA	12829.81	.048	.064	.085	643.855	22	< .001
Models comparison							
(1) vs. (2)		.024	.032	.042	319.037	16	< .001
(2) vs. (3)		.008	.010	.013	104.675	1	< .001

*Note*. AIC = Akaike information criterion; RMA = rape myth acceptance; *R*²_McF_ = McFadden’s pseudo-*R*²; *R*²_CS_ = Cox and Snell’s pseudo-*R*²; *R²*_N_ = Nagelkerke’s pseudo-*R*².

The model with only the country variable confirms the conclusions of Figure 1, distinguishing a group of three countries (Slovakia, Czechia, and Poland) where the probability of relative leniency toward partner rape is clearly higher with respect to Austria (each *p* < .001, OR from 1.873; 95% CI [1.641, 2.139] to 2.156 [1.875, 2.480]), but also, as the model coefficients indicate, in relation to Germany and Hungary. Hungary is an in-between case, with a slightly higher probability of lenient partner rape treatment than Austria (OR: 1.263 [1.097, 1.455], *z* = 1.973, *p* = .001).

The FULL model has a significantly higher information value than COUNTRY model (*χ*²(16) = 319.037, *p* < .001; pseudo-*R*^2^ values about 2.5 times larger, lower AIC). These new McFadden’s (.040) and Nagelkerke’s (.072) pseudo-*R*² values correspond to a rather low strength of the overall effect, see Table 3. This means that the model still does not explain much of the variance in relative leniency toward partner rape. The most significant variables in the full model are Country, Age, Sex, Religion and SDO (at least one coefficient significant with *p* < .001 for each), followed by RWA (*z* = 2.297, *p* = .022), and years of education are potentially significant (*p* = .045). The degree of urbanization is not significant (for coefficients *p* > .265, in the omnibus test *p* = .303). Typically, the probability of treating partner rape more leniently is higher in the 55 and older age group, especially 65 years and older (OR: 2.031 [1.708, 2.415], *p* < .001)—compared to the 18 to 24 group, which is not significantly different than other groups younger than 55 years. This probability is also higher in every religion except Orthodox (*p* = .135; the lack of significance of the effect may have been due to the small size of this group, *n* = 101), and especially among Muslims (OR: 1.732 [1.212, 2.477], *p* = .003). Both SDO and RWA positively correlate with the probability studied, but the effect of SDO was stronger and more significant (OR: 1.115 [1.065, 1.166], *p* < .001 vs. 1.055 [1.008, 1.104], *p* = .022). Years of education negatively correlate with explained probability, but the effect is very small and likely reaches significance due to the large sample size (OR: 0.957 [0.917, 0.999], *p* = .045). Although the variables gender, religion, SDO, and RWA are significant in the model, it should be noted that the strength of their effect is weak: OR < 1.4 in each case, corresponding to Cohen’s *d* < 0.2; the exception is the Muslim group, with an OR of about 1.7, which corresponds to a *d* of about 0.3 (Chinn, 2000).^4^ Meanwhile, the highest effect strengths are found for three countries and the oldest age group (OR of about 2, which corresponds to Cohen’s *d* of about 0.4), and while still not strong, should undoubtedly be considered substantively valid.

The full model only slightly reduced the impact of the Country variable (omnibus test *χ*²: 220.144 vs. 188.298), rendering the difference between Austria and Germany insignificant and slightly reducing the coefficient for Slovakia. According to the *χ*² omnibus test, however, the Country variable still carries the greatest information value in the model and allows us to distinguish a group of three countries where the occurrence of lenient treatment of partner rape is much more likely, but the information value of age groups is similarly high (*χ*²(5) = 188.298 for Country vs. *χ*²(5) = 173.318 for age).

### The Effect of Rape Myth Acceptance

Including Rape Myth Acceptance in the model significantly improved its fit (*χ*²(1) = 104.675, *p* < .001) and increased pseudo-*R*^2^ measures by around 20%, see Table 3. Rape Myth Acceptance has a strongly significant, albeit not such a strong positive correlation with the explained probability (OR: 1.314 [1.247, 1.385], *p* < .001) and was one of the three most important variables in the model (*χ*²(1) = 104.675 compared to *χ*²(5) = 175.940 for Age and *χ*²(5) = 143.478 for Country). Importantly, the addition of Rape Myth Acceptance significantly weakens the impact in the model of all previously significant variables except Age (see the comparison of *χ*² in omnibus test before and after Rape Myth Acceptance inclusion in Table 2), and in particular renders insignificant RWA (OR: 1.017 [0.971, 1.065], *p* = .475) and years of education (OR: 0.975 [0.933, 1.018], *p* = .244), while it leaves the SDO variable barely significant and having negligible effect (OR: 1.051 [1.002, 1.101], *p* = .040).

## Discussion

We have found some support for the overarching hypothesis that the rape of an intimate partner is seen as calling for less harsh punishment than that of a stranger. This bias holds true across all jurisdictions studied and in no major demography do we observe the opposite trend (i.e., consistent leniency toward stranger rape). Despite their identical legal status, the differential public punitiveness toward both offenses persists in each country studied here. The broad ultra-punitive turn against sexual offending (Pratt, 2000) has not eliminated disparities privileging partner rape. Those respondents who differentiated their punishment recommendations were much more likely to do so in favor of partner rape. In many groups though, equal sentences for stranger and partner rape were the single most common choice, meaning that a large part of Central European societies now recognizes the gravity of rape regardless of the victim-offender relationship.

As regards cross-national differences, the studied jurisdictions split into two easily distinguishable groups, with Germans, Austrians, and Hungarians less likely than their Slavic neighbors (Czechs, Poles, and Slovaks) to grant special leniency to partner rape. The composition of these groups is stable across models and country coefficients remain intact after the addition of further significant predictors. A glance at recent history helps make sense of this divide. Since the 1970s, the women’s rights movement in West Germany has been raising awareness about sexual violence in intimate relationships and the notion of “marital duties” was gradually losing popularity (Freeland, 2021). Boosted by cultural proximity, the same social forces were at work in Austria, which eventually outlawed spousal rape in 1989.

In contrast, the “rights revolution” and second-wave feminism simply did not happen in the people’s democracies east of the Iron Curtain (Šiklová, 1997; Stachniak, 1995). After the Berlin Wall fell, illiberal politics came to readily blame rape on sexual deviants and Middle-Eastern refugees, while partner rape was paved over in the name of “family values,” which were allegedly jeopardized by undue state intrusion into marital affairs. Czechia and Slovakia are now among the few European countries that have thus far failed to ratify the Istanbul Convention opposing violence against women and domestic abuse, and the Polish government in 2020 threatened to withdraw from the treaty, believing it to go against the country’s family traditions.^5^

Some (but not all) coefficients for demographic variables resonate with the literature on rape myths. Men, who are seen by some as the primary beneficiaries of these myths (Burt, 1980), proved relatively less punitive toward partner rape (cf. Ben-David & Schneider, 2005), but this effect was small. Differential punitiveness in the same direction was more common in the older cohorts of 55+, with a sharp increase past the age of 65; those socialized in the more patriarchal past might hold beliefs at odds with the modern understanding of sexual self-determination in romantic relationships. At the same time, the degree of urbanization was found not to be a significant predictor of differential punitiveness.

Conventional beliefs conducive to partner rape myths are often anchored in religious upbringing (Edwards et al., 2011). Our research shows that followers of all major denominations are more likely to recommend a lighter penalty for partner rape; however, the effect even among Muslims, where it was highest, remained weak. Forbearance toward partner rape may flow from the subordination of a woman to her husband, a common trope in Roman Catholicism, Protestantism, and Islam alike. These observations should be read together with empirical findings at the religion-rape myths nexus on the one hand (Barnett et al., 2018; Freymeyer, 1997), and interpretive analyses of misogynist teachings on the other hand (Fortune, 2005; Ozorak, 1996). That said, more liberal or egalitarian readings of the Bible and the Qur’an have also been in circulation, and it is not uncommon for modern religious leaders to condemn any form of intramarital abuse as a transgression against the spousal duty of care (e.g., Ali, 2004). The last point can explain rather weak effect of religion—much smaller than one that would be consistent with the hypothesis that religions are inextricably linked to oppression against women resulting in a strong bias in favor of partner rape.

Turning now to RWA and SDO, both scales predicted a probability of bias. Their independent effects are explained by the dual-process model of prejudice proposed by Duckitt (2001), according to whom the scales express two different motivational goals underlying conservative ideology. High RWAs prioritize in-group conformity and may perceive female victims of partner rape as threats to family values, fearing that their reports may disrupt familial stability. Conversely, SDO is associated with stereotypes against perceived weaker groups. This implies that the leniency toward partner—especially marital—rape could also stem from the belief that females should be subordinate to their partners, and thus less entitled to seek legal redress compared to victims of stranger rape. That said, it needs to be emphasized that the effects of RWA and SDO were much weaker than one would expect. This is an interesting finding, as it shows that while these constructs seem theoretically related to the bias under consideration, they cannot effectively capture the sources of such views.

The additional model showed that differential punitiveness in favor of the partner is more prevalent among individuals who subscribe to a set of beliefs known as rape myths, even the myths unrelated to the relationship between victim and perpetrator. As expected, bias in favor of partner rape appears to emanate from rape myth acceptance. Importantly, adding the rape myth acceptance to the model undermined the relevance of the RWA and SDO. Rape myth acceptance, a scale conceptually related to our outcome variable, might act as a more proximal cause of the observed bias, as it consists of judgments directly concerned with rape, while RWA and SDO reflect one’s worldview only on a general level.

The inclination to treat partner rape more leniently may also stem from cognitive dissonance. This occurs when the positive connotations of a romantic relationship—closeness, intimacy, and trust—clash with the negative connotations of rape—violence, and harm. Dissonance theory suggests that exposure to conflicting information causes unpleasant arousal, motivating individuals to resolve the dissonance (Festinger, 1957; Harmon-Jones & Mill, 1999). In cases of partner rape, this contradiction between the expected dynamics of a romantic relationship and the reality of rape may lead individuals—particularly those with traditional worldviews—to minimize the seriousness of the situation to align it with their existing beliefs about family and intimate relationships.

Sentiments like this seep into the daily practice of criminal justice systems and influence trial outcomes, as evidenced by quantitative and qualitative studies in the English-speaking world. One of the British judges interviewed by Krahé and Temkin (2008, p. 138) believed “without a shadow of doubt” that date rape is less serious, while another added: “When it’s the boyfriend, you’re probably not in fear of your life,” a bold claim to be made in a country where most femicides are committed by victims’ current or former partners (Femicide Census, 2020). A Canadian file study found victim-offender relationships to influence sentence length even when harm and violence were controlled for (McCormick et al., 1998). Our findings suggest that the same might apply to Central European courts, and future research can uncover the scale of such disparities.

## Diversity, Limitations, and Further Research

Alongside other research objectives, we attempted to extend the body of literature on how stranger and partner rape are perceived in Central Europe. While survey data allowed us to study populations beyond student samples, we acknowledge that people with marginalized identities might have been less likely to participate in online panels.

The survey items did not specify the incident beyond classifying it as a first-time offense and a stranger or partner rape. Respondents were thus free to picture the incident according to stereotypes and heuristics (Stalans, 2002), which we consider inherent in attitudes toward stranger and partner rape in general as they appear in the public debate. However, this means that we cannot distinguish between the direct and indirect impact of the victim-offender relationship on punishment recommendations. Some respondents could have chosen shorter sentences for partner rape thinking that the intimate relationship alone should be a mitigating factor. Others, in turn, could have arrived at the same sentencing disparity simply by imagining one incident as more violent than the other. Partner rape myths may, thus, have both direct and indirect influences, but future vignette-based research could determine their relative impacts on punishment outcomes. The rather small measures of variance explained suggest that these and other unmeasured factors could be operative, alongside large noise characteristic of ambiguous punishment preferences (Uhl & Pickett, 2025). Finally, it is possible that the decision-making factors that lead to the differential punitiveness of sex crimes are so complex that they cannot be explained effectively without the skillful application of qualitative methods.

## Conclusion

Our study extended the findings on differential punitiveness in the general public in six countries, showing that bias in favor of partner rape is more common than bias in favor of stranger rape. In the rare instances when the latter occurs, the disparity is typically smaller. Differential punitiveness was not evenly distributed among jurisdictions and other demographics. Interestingly, we found that many predictors of overall punitiveness, such as country, religion, and age (see Gerber, 2021 for review) are also predictive of differential punitiveness toward the two instances of sex offending studied. The punitive sentiment toward rape remains partly dependent on the relationship between perpetrator and victim, which reflects the role of gender stereotypes and rape myths in shaping the punishment preferences of the public; the exact reasons for this sentiment are still to be elucidated.

## Notes

1. We use the term “bias” as a shorthand for differential punitiveness in favor of one type of rape despite no legally relevant difference between the two; in the text, we refer mainly to bias in favor of partner rape compared to stranger rape.

2. However, Munge et al.’s (2007) respondents viewed marital rape as more traumatizing for the victim when the couple had been married for longer.

3. The remaining items were: “When men rape, it is usually because of their strong desire for sex” (excluded due to low factor loading and a negative impact on reliability, i.e., worsening Cronbach’s α by ca. .05). “It shouldn’t be considered rape if a man is drunk and didn’t realize what he was doing.” “If a woman doesn’t physically resist sex—even if protesting verbally—it can’t be considered rape.” “A lot of times, women who say they were raped often led the man on and then had regrets.”

4. Odds ratio can be converted to approximated Cohen’s *d* effect size by dividing OR natural logarithm by π/√3, which is about 1.81 (Chinn, 2000).

5. However, all of the above applies to Hungary, a peculiar case with the highest prevalence of the bias in favor of stranger rape.
